# Cross-Domain Object Detection with Hierarchical Multi-Scale Domain Adaptive YOLO

**DOI:** 10.3390/s25175363

**Published:** 2025-08-29

**Authors:** Sihan Zhu, Peipei Zhu, Yuan Wu, Wensheng Qiao

**Affiliations:** National Key Laboratory of Complex Aviation System Simulation, Southwest China Institute of Electronic Technology, Chengdu 610036, China; zhupeipei0806@sina.com (P.Z.); wuyuaner@126.com (Y.W.); wshqiao0529@163.com (W.Q.)

**Keywords:** object detection, domain adaptation, hierarchical, multi-scale, YOLO

## Abstract

To alleviate the performance degradation caused by domain shift, domain adaptive object detection (DAOD) has achieved compelling success in recent years. DAOD aims to improve the model’s detection performance on the target domain by reducing the distribution discrepancy between different domains. However, most existing methods are built on two-stage Faster RCNN, which is not suitable for real applications due to the detection efficiency. In this paper, we propose a novel Hierarchical Multi-scale Domain Adaptive (HMDA) method by integrating a simple but effective one-stage YOLO framework. HMDAYOLO mainly consists of the hierarchical backbone adaptation and the multi-scale head adaptation. The former performs hierarchical adaptation based on the differences in representational information of features at different depths of the backbone network, which promotes comprehensive distribution alignment and suppresses the negative transfer. The latter makes full use of the rich discriminative information in the feature maps to be detected for multi-scale adaptation. Additionally, it can reduce local instance divergence and ensure the model’s multi-scale detection capability. In this way, HMDA can improve the model’s generalization ability while ensuring its discriminating capability. We empirically verify the effectiveness of our method on four cross-domain object detection scenarios, comprising different domain shifts. Experimental results and analyses demonstrate that HMDA-YOLO can achieve competitive performance with real-time detection efficiency.

## 1. Introduction

Object detection is a core technique in the field of computer vision, which aims to recognize and localize objects of interest in images or videos. And it can help individuals to extract key information from complex visual environments. Traditional object detection algorithms rely on hand-crafted features, with drawbacks, such as high computational costs, poor robustness, and low accuracy. In recent years, deep learning has gained popularity [1], which can learn more robust and generalizable deep feature representations. Specifically, object detection has made impressive progress thanks to convolutional neural networks (CNNs). And it has become an essential part of many real-world applications. The mainstream three detectors are Faster RCNN [2], You Only Look Once (YOLO) [3], and Single Shot Multi-box Detector (SSD) [4]. However, the superior performance of advance detectors relies on the availability of large number of high-quality annotated data. Acquiring such data is time consuming and labour intensive. Additionally, well-trained detectors may experience a sudden drop in performance due to the domain shift problem when processing new data or tasks.

The aforementioned problems seriously affect the application and deployment of the detection models when data distributions are different. Unsupervised Domain Adaptation (UDA) [5,6,7] has been proposed to deal with the domain shift problems and reduce the dependence of model training on target labels. It aims to transfer the source knowledge to the target and reduce the distribution discrepancy between different domains, which enhances the model’s generalization ability and discriminative capability. Deep UDA methods can be divided into two categories: Moment matching methods [8,9,10] explicitly match the feature distribution across domains during network training based on pre-defined metrics. Adversarial learning methods [11,12,13] implicitly learn domain-invariant representations in an adversarial paradigm.

Unlike classification and semantic segmentation, the object detection task predicts bounding box localization and corresponding object category [14]. This brings potential problems and challenges for cross-domain object detection, but has likewise raised a lot of concerns. Recent studies have made significant efforts to improve the cross-domain detection capabilities. Following the first try on cross-domain object detection, domain adaptive Faster RCNN [15], most existing DAOD methods are still built on the two-stage detector Faster RCNN [16,17,18,19,20,21,22,23]. Few works have utilized one-stage detectors (e.g., SSD [4] and FCOS [24]) to consider the computational efficiency [25,26,27,28]. And some methods have also been proposed for light-weight and practical use based on the YOLO series [29,30,31,32]. Overall, the development of DAOD methods is closely related to the key technologies in the field of object detection and domain adaptation.

Since the scene layout, number of objects, patterns between objects, and the background may be quite different across domains in object detection tasks, a potential problem in DAOD tasks is that blindly and directly adapting feature distributions can lead to negative transfer, which degrades the model’s cross-domain performance. This is also the reason why strategies like prototype alignment and entropy regularization, which work well in image classification or segmentation, become inapplicable in object detection. In addition, some of the methods utilize image generation techniques to introduce auxiliary data [22,31], or adopt a student–teacher network paradigm [32,33] for training. The former makes the training process not an end-to-end mode, and the latter significantly increases the model’s complexity and the difficulty of the training model. These types of methods greatly restrict the training and application scenarios of detectors. Finally, the efficiency and accuracy of the dominant Faster RCNN are outdated, which is not suitable for resource-limited and time-critical real applications [32].

For the above reasons, we propose a novel Hierarchical Multi-scale Domain Adaptive method, HMDA-YOLO, based on the simple but effective one-stage YOLOv5 [34] detector. HMDA-YOLO is easy to implement and has competitive performance, which consists of the hierarchical backbone adaptation and the multi-scale head adaptation. Considering the differences in representation information of features at various depths, we designed the hierarchical backbone adaptation strategy, which promotes comprehensive distribution alignment and suppresses the negative transfer. Specifically, we adopt the pixel-level adaptation, image-level adaptation, and weighted image-level adaptation for the adversarial training of shallow-level, middle-level, and deep-level feature maps, respectively. To make full use of the rich discriminative information of the feature maps to be detected, we further designed the multi-scale head adaptation strategy. It performs pixel-level adaptation across each detection scale, which reduces local instance discrepancy and the impact of background noise. Note that “pixel-level” denotes each location at the corresponding feature map and the “image-level” treats the entire feature map as a whole in this paper. The proposed HMDA-YOLO can significantly improve the model’s cross-domain capability. We empirically verify the HMDA-YOLO on four cross-domain object detection scenarios, comprising different domain shifts. Experimental results and analyses demonstrate that HMDA-YOLO can achieve competitive performance with high detection efficiency.

The contributions of this paper can be summarized as follows: (1) we propose a simple but effective DAOD method, HMDA-YOLO, for more accurate and efficient cross-domain detection; (2) a hierarchical adaptation strategy for the backbone network as well as a multi-scale adaptation strategy for the head network are designed to simultaneously ensure the model’s generalization and discriminating capability; (3) HMDA-YOLO can achieve competitive performance on several cross-domain object detection benchmarks, compared with state-of-the-art DAOD methods.

The remainder of this paper is organized as follows: Section 2 reviews the techniques related to DAOD. Section 3 explores the technical details of HMDA. Section 4 presents the experimental results and analysis. Finally, Section 5 provides a summary.

## 2. Related Work

### 2.1. Object Detection

The mainstream methods for object detection can be categorized into two-stage (e.g., Faster RCNN [2] and Mask RCNN [35]) and one-stage (e.g., SSD [4], FCOS [24], and YOLO series [3]). Faster RCNN [2] is the representative method for two-stage detectors. Its main contribution is the region proposal network (RPN), and it integrates feature extraction, proposal detection, bounding box regression, and classification into a single unified framework. SSD [4] predicts bounding box and category from feature maps at different scales for fast and accurate object detection. The YOLO series has undergone several version upgrades. YOLO [3] first regards the detection task as regression and it can realize real-time detection. YOLOv3 [36] utilizes Darknet-53 as the backbone network and can obtain multi-scale predictions by adding the Spatial Pyramid Pooling (SPP) module. YOLOv4 [37] examines the contribution of different tricks on the object detection task and extends the Darknet-53 backbone with Cross Stage Partial (CSP) connection. YOLOv5 [34] modifies the CSPDarknet-53 and makes use of multiple augmentation techniques for faster and better capability. In recent years, DEtection TRansformer (DETR) [38] shows competitive performance, which treats object detection as a set prediction task.

### 2.2. Unsupervised Domain Adaptation

As mentioned before, there are two mainstream methods of UDA. Moment matching methods usually measure the distribution discrepancy by a pre-defined metric (e.g., Maximum Mean Discrepancy), and explicitly match the feature distribution across domains by reducing such discrepancy during network training. The Deep Adaptation Network (DAN) [8] extends the maximum mean discrepancy with multi-kernel and optimizes it with multiple layers. The Joint Adaptation Network (JAN) [9] tries to simultaneously match the margin and conditional distribution. The Deep Sub-domain Adaptation Network (DSAN) [10] aligns sub-domains for fine-grained distribution matching. Adversarial learning methods extend the network with a domain discriminator or more classifiers, and implicitly learn domain-invariant representations in an adversarial paradigm. The Domain Adversarial Neural Network (DANN) [11] firstly combines adversarial training with domain adaptation. And it proposes the gradient reverse layer, which is the core of most adversarial methods. The Conditional Domain Adversarial Network (CDAN) [12] proposes multi-linear conditioning to make use of discriminative information for better adversarial training. The Dynamic Adversarial Adaptation Network (DAAN) [13] implements adversarial training in each category and dynamically adapts their weights.

### 2.3. Domain Adaptive Object Detection

Object detection is a fundamental task in the field of computer vision and has a wide range of real-world applications. Therefore, researchers pay more and more attention to cross-domain detection techniques [14]. As mentioned earlier, the Faster RCNN-based methods are currently the dominant. Domain Adaptive Faster RCNN (DAF) [15] is the first work to address the DAOD task by designing the image-level and instance-level adaptation. Strong-Weak Distribution Alignment (SWDA) [16] adjusts the adaptation balance between different network layers to avoid potential risks of blind transfer. Reference [18] used a domain randomization technique to generate source domains with different domain shifts, and unified multiple source domains to learn domain-invariant features by representation learning. Reference [22] proposed the Hierarchical Transferability Calibration Network (HTCN) to improve transferability while maintaining discriminability, which extends existing adaptation strategies with image-to-image translation.

Comparatively, DAOD approaches based on one-stage detectors do not obtain enough attention. Reference [25] enhanced the SSD framework with weakly supervised learning for cross-domain detection. Reference [26] used the style transfer and pseudo-labeling approach for pixel-level adaptation and noise reduction. Reference [27] proposed an Implicit Instance-Invariant Network (I^3^Net) to learn instance-invariant features by designing multiple strategies and modules. Specifically, domain-adaptive YOLO architectures are proposed for resource-limited and time-critical real applications. References [29,30] extended YOLOv3 and YOLOv4 at different scales to learn domain-invariant features, respectively. Stepwise Domain Adaptive YOLO (S-DAYOLO) framework [31] was proposed to bridge the domain gap by constructing an auxiliary domain for autonomous driving. Semi-supervised Domain Adaptive YOLO (SSDA-YOLO) [32] combines the knowledge distillation framework and scene style transfer with the mean teacher model, and further proposes a consistency loss for distribution alignment based on the YOLOv5.

It is worth noting that with the development of DETR detectors, related cross-domain methods have also received widespread attention. Sequence Feature Alignment (SFA) designs a domain query-based feature alignment module and a token-wise feature alignment module for the adaptation of detection transformers [39]. The Domain Adaptive Detection Transformer (DA-DETR) introduces information fusion for effective knowledge transfer [40].

As mentioned earlier, there are numerous DAOD algorithms built on the outdated Faster RCNN. In this paper, we select the lightweight YOLO framework, which greatly improves inference speed while ensuring feature extraction capability. By comprehensively aligning the features in the backbone and head network, HMDA not only effectively enhances the model’s generalization ability but also avoids the degradation of target discriminability. It is shown that, combined with the proposed hierarchical multi-scale domain adaptation approach, very competitive results on various cross-domain detection scenarios can be obtained even by the small version of the YOLO framework.

## 3. Method

**Figure 1:**
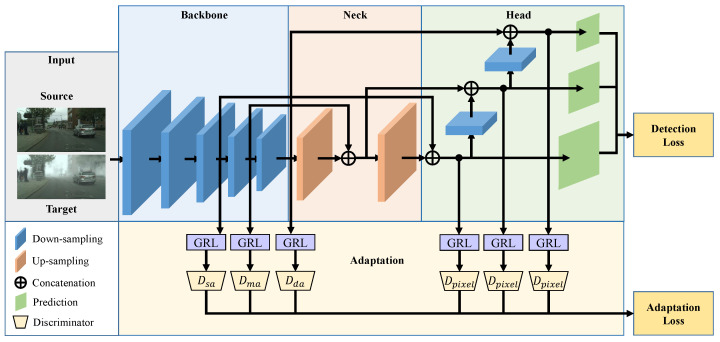
The overall structure of HMDA-YOLO. It mainly consists of two main parts, the backbone adaptation and the head adaptation. The hierarchical adaptation of the feature distribution in the backbone part of YOLOv5 framework alleviates the affection of negative transfer and makes the adaptation more comprehensive. The multi-scale head adaptation significantly reduces local instance discrepancy and the impact of background noise. In this way, HMDA enhances the model’s cross-domain performance and not impairs its discriminative ability, which enables real-time and accurate detection across domains.

In this section, we introduce the technical details of the proposed HMDA. An overview of the HMDA based on YOLOv5 architecture is shown in Figure 1. HMDA-YOLO hierarchically extracts the deep features of the image and performs specific adaptations at different depths by embedding multiple domain discriminators, which are distributed in the backbone network and the head network of YOLOv5. More information is described below.

### 3.1. Preliminary Knowledge

We will briefly introduce the YOLOv5 framework. YOLOv5 is a simple but effective one-stage detector and has been widely used in real-world applications. It mainly contains three parts, which is shown in the top part of Figure 1. (1) The backbone network is responsible for deep feature extraction. The CSPDarknet53 [37] is the most commonly used backbone network, composed of convolutional module, C3 module, and the Spatial Pyramid Pooling (SPP) module [41]; (2) The neck network further processes the extracted features and performs feature fusion to enhance the feature representative capability. It can achieve top-down and bottom-up feature fusion by the utilization of the Feature Pyramid Network (FPN) [42] and Path Aggregation Network (PAN) [43]; (3) The head network implements multi-scale detection of small, medium, and large objects.

In the cross-domain object detection tasks, there are two domains: a labeled source domain with $n_{s}$ images, denoted as $D_{S}={\left\{x_{i}^{s},b_{i}^{s},y_{i}^{s}\right\}}_{i=1}^{n_{s}}$, where $x_{i}^{s}$ denotes the source image, $b_{i}^{s}$ is the coordinate of bounding box, and $y_{i}^{s}$ is the object category. An unlabeled target domain with $n_{t}$ images, denoted as $D_{T}={\left\{x_{i}^{t}\right\}}_{j=1}^{n_{t}}$. The label space of the source and target domains are identical, but their data distributions are different. The goal of domain adaptive object detection is to train a detector to reduce the domain shift and learn transferable representations that the model can generalize well to the target objects.

### 3.2. Hierarchical Multi-Scale Adaptation

The adversarial DAOD methods usually learn domain-invariant feature representations with the help of the domain discriminator (i.e., domain classifier). Assuming that the framework is composed of a feature extractor *F* and a domain discriminator *G*. The feature extractor *F* tries to confuse the domain discriminator *G*, which means maximizing the domain classification loss. Conversely, the domain discriminator *D* is trained to distinguish the source samples from the target samples, which means minimizing the domain classification loss. The feature extractor *F* also learns to minimize the source supervision loss. The adversarial loss can be written as follows:

(1) $$\max_{F}\min_{D\in H}\left\{E_{D_{S}}+E_{D_{T}}\right\},$$

$E_{D_{S}}$ and $E_{D_{T}}$ denote the expected domain classification error over the source and target domain, respectively. By utilizing the Gradient Reverse Layer (GRL) [11], the min–max adversarial training can be unified in one back-propagation.

For object detection tasks, each activation on the feature map corresponds to a patch of the input image, and detectors will perform classification and regression on each location. Therefore, the domain discriminator can implement not only image-level distribution alignment, but also pixel-level distribution alignment. Since HMDA-YOLO hierarchically aligns the feature distribution in the backbone network and the head network, we will focus on these two parts.

#### 3.2.1. Hierarchical Backbone Adaptation

The backbone network hierarchically adapts the output features of each C3 module (i.e., the layer 4, 6, and 9 in YOLOv5 framework) through different ways, according to the representation information of the features at different depths.

The shallow feature can capture a lot of detailed information (e.g., edges, colors, textures, and angles), which not only facilitates the detection of small objects, but also helps the alignment of local features of cross-domain objects. According to [44], the least-square loss function can stabilize the training process of the domain discriminator and have advantages on aligning shallow representations. Therefore, we use it as the criterion to implement shallow-level feature adaptation. The domain discriminator $D_{sa}$ is a fully convolutional network with 1×1 kernel to predict the pixel-level domain label of the source and target feature maps. The shallow-level adaptation loss can be formulated as follows:

(2) $$L_{sa}=\sum_{i,u,v}D_{sa}{\left(f_{i}^{s}\right)}_{u,v}^{2}+\sum_{i,u,v}{\left(1-D_{sa}{\left(f_{i}^{t}\right)}_{u,v}\right)}^{2}$$

where $f_{i}^{s}$ and $f_{i}^{t}$ are the source and target shallow-level feature map of the input image $x_{i}$, respectively. And $D_{sa}{\left(f_{i}\right)}_{u,v}$ is the domain prediction in location (u,v) of the corresponding feature map.

The medium-level feature contains information about localized shapes and simple objects. And the Binary Cross Entropy (BCE) loss is used for adversarial loss calculation. The domain discriminator $D_{ma}$ is different from $D_{sa}$ that $D_{ma}$ treats each feature map as a whole and predicts image-level domain label. Specifically, $D_{ma}$ is composed of common convolutional layers, a global average pooling layer, and a fully connected layer. The middle-level adaptation loss can be formulated as follows:

(3) $$L_{ma}=-\sum_{i}\left[d_{i}\log D_{ma}\left(f_{i}^{m}\right)+\left(1-d_{i}\right)\log\left(1-D_{ma}\left(f_{i}^{m}\right)\right)\right],$$

where $f_{i}^{m}$ is the medium-level feature map of the input image $x_{i}$. And $D_{ma}\left(f_{i}^{m}\right)$ is the image-level domain prediction of $x_{i}$. $d_{i}$ is the ground truth domain label, which is 0 for the source domain and 1 for the target domain in this paper.

The scene layout, number of objects, patterns between objects, and the background may be quite different across domains. According to [16], it is likely to hurt performance for larger shifts. As an example, the source domain that contains rural images and the target domain that contains urban images may have large domain discrepancy, even if they share the same object category. In this case, blind alignment may lead to the negative transfer and impair the model’s capability between different domains. HMDA-YOLO alleviates the above problem by adjusting the weights of hard-to-classify and easy-to-classify samples to more robustly improve the model’s cross-domain performance. Specifically, for the deep-level feature adaptation, the BCE loss is extended with focal loss [45] to re-weight different samples. The domain discriminator $D_{da}$ is trained to distinguish the source from the target, which is similar to $D_{ma}$. The deep-level adaptation loss can be written as follows:

(4) $$L_{da}=-\sum_{i}\left[d_{i}{\left(1-D_{da}\left(f_{i}^{d}\right)\right)}^{\gamma }\log D_{da}\left(f_{i}^{d}\right)+\left(1-d_{i}\right)D_{da}{\left(f_{i}^{d}\right)}^{\gamma }\log\left(1-D_{da}\left(f_{i}^{d}\right)\right)\right],$$

where $f_{i}^{d}$ is the deep-level feature map of the input image $x_{i}$. γ is the parameter to control the weight of different samples. If a sample is easy-to-classify (i.e., far from the decision boundary), it is desired to have a low loss to avoid negative transfer. Conversely, if it is hard-to-classify, we want it to have a high loss for domain confusion. Therefore, the value of γ needs to be greater than 1 to assign low weight for the easy-to-classify samples and high weight for the hard-to-classify samples.

Combining the hierarchical domain adaptation loss, HMDA-YOLO promotes comprehensive distribution alignment and suppresses the negative transfer. The overall adaptation loss function of the backbone network can be written as follows:

(5) $$L_{backbone}=L_{sa}+L_{ma}+L_{da}.$$

#### 3.2.2. Multi-Scale Head Adaptation

The feature maps of the head network play an important role in multi-scale object detection, which are crucial for object recognition and localization. Therefore, HMDA-YOLO proposes to implement fine-grained pixel-level adaptation at each scale for efficient cross-domain training, based on the rich discriminative information of the feature maps to be detected. On the one hand, multi-scale adaptation can reduce the local instance divergence and guarantee the model’s multi-scale detection capability. On the other hand, it helps to distinguish between the object and the background, which reduces the impact of background noise. In this way, the model can learn more robust and generalizable representations to improve the detection accuracy.

Specifically, the head adaptation is performed at three different scales, i.e., layer 17, 20, and 23 in YOLOv5 framework. The pixel-level domain discriminator $D_{pixel}$ is similar to $D_{sa}$, which predicts pixel-level domain labels. And BCE loss function is used for loss calculation. The adaptation loss at each scale can be written as follows:

(6) $$L_{pixel}=-\sum_{i,u,v}\left[d_{i}\log D_{pixel}{\left(f_{i}\right)}_{u,v}+\left(1-d_{i}\right)\log\left(1-D_{pixel}{\left(f_{i}\right)}_{u,v}\right)\right],$$

where $f_{i}$ denotes the feature map to be detected at each scale. Note that $D_{pixel}$ in three different scales do not share the weight. Considering all three scales feature adaptation, the head adaptation loss can be unified as follows:

(7) $$L_{head}=\sum_{s=1}^{3}L_{pixel}^{s}$$

where *s* denotes different scales.

### 3.3. Overall Formulation

The main discriminative capability of the network is learned from the source labeled samples in supervised way. And the source supervised training loss of YOLOv5 can be formulated as follows:

(8) $$L_{det}\left(X^{s},B^{s},Y^{s}\right)=L_{box}\left(B^{s};X^{s}\right)+L_{cls}\left(Y^{s};X^{s}\right)+L_{obj}\left(Y^{s};X^{s}\right),$$

where $X^{s}$, $B^{s}$, and $Y^{s}$ denote the set of $x_{i}^{s}$, $b_{i}^{s}$, and $y_{i}^{s}$. $L_{cls}$ is the classification loss and defaults to the BCE loss. $L_{obj}$ is the object confidence loss and defaults to the BCE loss. $L_{box}$ is the bounding box regression loss and defaults to the CIoU loss.

The domain adversarial loss combined with the hierarchical backbone adaptation strategy and the multi-scale head adaptation strategy can be written as follows:

(9) $$L_{adv}=L_{backbone}+L_{head}.$$

**Figure 2:**
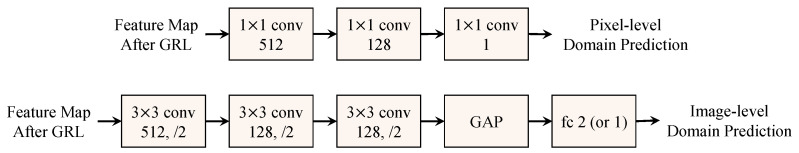
The structure of two types of domain discriminator. Top: pixel-level domain discriminator. Bottom: image-level domain discriminator. GAP means global average pooling layer. The information in the convolutional layer indicates the size of the convolutional kernels, the number of output channels, and whether or not to downsample, respectively.

Combining the source supervised loss function with the cross-domain adversarial loss function, the overall optimization objective can be formulated:

(10) $$\max_{D}\min_{G}L_{det}-\lambda L_{adv},$$

where *D* denotes the set of all domain discriminators, including $D_{sa},D_{ma},D_{da}$, and $D_{pixel}$. λ is the trade-off parameter to balance the detection loss and the domain adversarial loss. The network can be trained in an end-to-end manner using a standard stochastic gradient descent algorithm. And the adversarial training can be achieved by the utilization of GRL, which reverses the gradient during propagation. Structures of these domain discriminators are summarized in Figure 2.

### 3.4. Theoretical Analysis

Reference [46] designed HΔH-distance to measure the divergence between two sets of samples that have different data distributions. Let us consider a source domain $D_{S}$, a target domain $D_{T}$, and a domain discriminator h:x↦{0,1}, which tries to predict the source and target domain label to be 0 and 1, respectively. Assuming that H to be a set of possible domain discriminators, the HΔH-distance can be defined as follows:

(11) $$d_{H\Delta H}\left(D_{S},D_{T}\right)=2\underset{h,h^{\prime }\in H}{\sup}\left|E_{x∼D_{S}}\left[h\left(x\right)\neq h^{\prime }\left(x\right)\right]-E_{x∼D_{T}}\left[h\left(x\right)\neq h^{\prime }\left(x\right)\right]\right|,$$

where $E_{x∼D_{S}}$ and $E_{x∼D_{T}}$ denote the expected domain classification errors over the source domain and the target domain, respectively. The combined error of the ideal hypothesis (e.g., domain discriminator) can be denoted as follows:

(12) $$\begin{array}{cc} \lambda & =\epsilon_{S}\left(h^{*}\right)+\epsilon_{T}\left(h^{*}\right),\mathrm{where}\\ h^{*} & =\underset{h\in H}{\arg\min}\epsilon_{S}\left(h\right)+\epsilon_{T}\left(h\right) \end{array}$$

where $h^{*}$ is the ideal joint hypothesis. The terms $\epsilon_{S}\left(\cdot \right)$ and $\epsilon_{T}\left(\cdot \right)$ are the expected risks on source and target domains, respectively. It can be used to measure the adaptability between different domains. If the ideal joint hypothesis performs poorly, i.e., the error λ is large, the domain adaptation process is difficult to realize. Based on the above knowledge, Reference [46] gives a upper bound on the target error as follows:

(13) $$\forall h\in H,\;\epsilon_{T}\left(h\right)\leq \epsilon_{S}\left(h\right)+\frac{1}{2}d_{H\Delta H}\left(D_{S},D_{T}\right)+Const,$$

the target error is upper bounded by three terms, including the expected error on the source domain $\epsilon_{S}\left(h\right)$, the domain divergence $d_{H\Delta H}\left(D_{S},D_{T}\right)$, and few constant terms. Since the $\epsilon_{S}\left(h\right)$ can be directly minimized by the network supervised learning and the third term is difficult to handle, with the majority of existing methods minimize the upper bound on the target error by reducing the domain divergence between source and target domains. Our proposed method not only hierarchically optimizes the distribution discrepancy between different domains at the backbone network, but also alignment the multi-scale features before the detection in the head network. Thus, it can effectively reduce the distribution discrepancy and significantly improve the cross-domain detection performance.

## 4. Experiment

In this section, we verify the proposed HMDA-YOLO on different cross-domain object detection tasks. Related datasets consist of Cityscapes, Foggy Cityscapes, Sim10k, KAIST, Pascal VOC, and Clipart. Quantitative results and related analyses demonstrate the superiority of the proposed HMDA-YOLO.

### 4.1. Datasets

Cityscapes [47] is a large-scale street scene dataset for driving scenarios. The images are captured by a car-mounted video camera from 50 different cities. It consists 2975 images in the training set and 500 images in the validation set.

Foggy Cityscapes [48] dataset is rendered from Cityscapes by adding synthetic fog to real, clear-weather images using incomplete depth information. The data split and semantic annotations of Foggy Cityscapes are inherited from Cityscapes.

Sim10k [49] dataset is generated by the game engine of Grand Theft Auto V (GTA V). It contains 10,000 images of synthetic driving scene with 58,701 bounding boxes of car for training.

KAIST [50] dataset has both RGB and long-wavelength infrared (LWIR) images for multi-spectral pedestrian detection and multi-source fusion. Since successive frames of images are similar, we refined it with 7373 images for training and 1229 images for testing. All the three categories are integrated as *person*.

Pascal VOC [51] is a realistic scenes dataset for segmentation and detection. The train-val datasets of Pascal VOC 2007 and 2012 are utilized together. And it consists of 16,551 images with 20 distinct object categories.

Clipart [25], which is a graphical image dataset with complex backgrounds, has the same 20 object categories with Pascal VOC. Specifically, it has 500 images for training and 500 images for testing.

We evaluate our method in the following scenarios: adaptation across adverse weather (Cityscapes → Foggy Cityscapes), adaptation from synthetic to real (Sim10k → Cityscapes), adaptation across heterogeneous data (KAIST RGB → KAIST LWIR), and adaptation across large domain shift (Pascal VOC → Clipart). More details of the datasets can be found in Table 1. C, F, S, KR, KL, P, and CL denote Cityscapes, Foggy Cityscapes, Sim10K, KAIST RGB, KAIST LWIR, Pascal VOC, and Clipart, respectively.

### 4.2. Experiment Setup

All experiments are implemented based on the PyTorch framework with a NVIDIA A800 GPU (TSMC, Hsinchu, Taiwan). And we choose YOLO framework with small parameters (YOLOv5s and YOLOv8s) as the base detector. All training and testing images are resized in the shape of 960×960. The stochastic gradient descent (SGD) is utilized for optimizing the model, where the learning rate is 0.01, weight decay is 0.0005 and momentum is 0.937. The batch size is set to 32 with 200 training epochs during the training stage. Other settings can be referred to [32]. For each cross-domain detection tasks, we report the average precisions (AP, %) and mean average precisions (mAP, %) with a threshold of 0.5. The tradeoff parameter λ is set to 1.0 without specified.

We compare our proposed HMDA-YOLO with some state-of-the-art DAOD methods, including Faster RCNN [2], YOLOv5s, YOLOv8s [34], DAF [15], SWDA [16], HTCN [22], ATF [52], UMT [33], TDD [53], TIA [54], Deformable-DETR [55], SFA [39], DA-DETR [40], S-DAYOLO [31] and ConfMix [56]. Faster RCNN, D-DETR, YOLOv5s, and YOLOv8s means the model is only trained with source samples (source only) and it then inferences directly on the target domain samples. Bold in each table indicates the optimal result, except for YOLOv8s.

### 4.3. Quantitative Results

#### 4.3.1. Adaptation Across Different Visibility

Differences in data distribution due to weather changes are very common in real-world applications, e.g., autonomous driving. The ability of the detection model to adapt to different weather is crucial. Therefore, we firstly evaluate our proposed HMDA-YOLO across adverse weather. Specifically, the Cityscapes dataset with clear weather and the Foggy Cityscapes with fog are used in this task. The quantitative results are shown in Table 2. Methods based on different detectors are separated in each table. It can be seen that the proposed HMDA-YOLO outperforms all the compared methods and achieves 45.9% mAP. And it is 2.8% higher than the second best TDD. HMDA-YOLO has an absolute mAP improvement of 19.6% and a relative improvement of 74.5% over the baseline YOLOv5s. Through the hierarchical backbone adaptation and multi-scale head adaptation, the affection of adverse weather on the model’s discriminative ability can be significantly mitigated. Additionally, HMDA-YOLO is extremely efficient for knowledge transfer on multiple categories, including car, mbike, bus, and train.

#### 4.3.2. Adaptation from Synthetic to Real

With rapid development of game engine and simulation software, we have easy access to a large number of lifelike scene images. Therefore, we can try to improve the model’s generalization ability with the help of synthetic images, by building cross domain models from synthetic to real. In particular, we employ synthetic Sim10K and real Cityscapes to compose this cross-domain task, with one category car to be detected. Detection results are shown in Table 3. Note that λ is set to 0.1 to obtain better performance according existing studies [16,22]. Our method achieves the best accuracy of 58.6%. And it outperforms the baseline YOLOv5s, UMT, and DA-DETR by 7.0%, 15.5%, and 3.9%. Note that all methods based on Faster RCNN perform poorly in this task, even lower than the yolov5s without adaptation.

#### 4.3.3. Adaptation Across Heterogeneous Data

There are many heterogeneous sensors that can acquire multiple types of heterogeneous data, such as RGB images, infrared images, and multi-spectral images. And the scenario of heterogeneous data adaptation is often ignored in DAOD research. We validate whether the proposed HMDA-YOLO can deal with heterogeneous data adaptation. The RGB and long-wavelength infrared (LWIR) images in the KAIST dataset are used as the source and target domains, respectively. The results are reported in Table 4. It can be seen that the YOLOv5s model trained with RGB images is completely unable to detect the person in LWIR images. And HMDA-YOLO can drastically improve the cross-domain performance of the model and achieves the accuracy of 45.4%, which demonstrates that the HMDA framework can efficiently implement the knowledge transfer even when the data are heterogeneous.

#### 4.3.4. Adaptation Across Large Domain Shift

Finally, we evaluate the proposed method on real-to-artistic adaptation datasets from Pascal VOC to Clipart. In this task, the source and target domains have large domain shift. The results of Pascal VOC to Clipart are shown in Table 5. The proposed HMDA-YOLO do not obtain the best performance with the accuracy of 39.1%. However, it still achieves the performance close to the SOTA methods. It is worth noting that it has a huge improvement of 18.7% over the baseline YOLOv5s (91.7% relative improvement), which also proves its superiority.

#### 4.3.5. Flexibility on the YOLO Series

The bottom of Table 2, Table 3, Table 4 and Table 5 present the quantitative results of YOLOv8s and HMDA based on it. The shallow-level, middle-level, deep-level, and multi-scale feature maps of YOLOv8 are layers 4, 6, 9, 15, 18, and 21, respectively. It can be seen that YOLOv8s has a stronger feature learning ability compared with YOLOv5s, and its source only model generally performs better than YOLOv5s. Moreover, the proposed HMDA can be flexibly integrated into other YOLO frameworks. HMDA based on YOLOv8s can obtain the accuracy of 54.4%, 64.8%, 33.8%, and 42.8% on task C→F, S→C, KR→KL, and P→CL, respectively. And it achieves the best results in tasks C→F, S→C, and P→CL, which demonstrates the flexibility and effectiveness of the proposed HMDA.

### 4.4. Analysis

#### 4.4.1. Ablation Study

To evaluate the contribution of different adaptation strategies, we recorded the ablation results in Table 6. There are 3 findings as follows: (1) Each component of our proposed HMDA-YOLO positively impacts the model’s cross-domain detection capability. (2) The hierarchical adaptation of the backbone network is more effective than the multi-scale adaptation of head network, especially the medium-level feature adaptation. (3) Combined with backbone and head adaptation strategy, our proposed HMDA-YOLO can achieve advanced performance. It demonstrate that HMDA-YOLO not only improves the generalization capability, but also guaranties the discriminative ability of the model.

#### 4.4.2. Detection Examples

**Figure 3:**
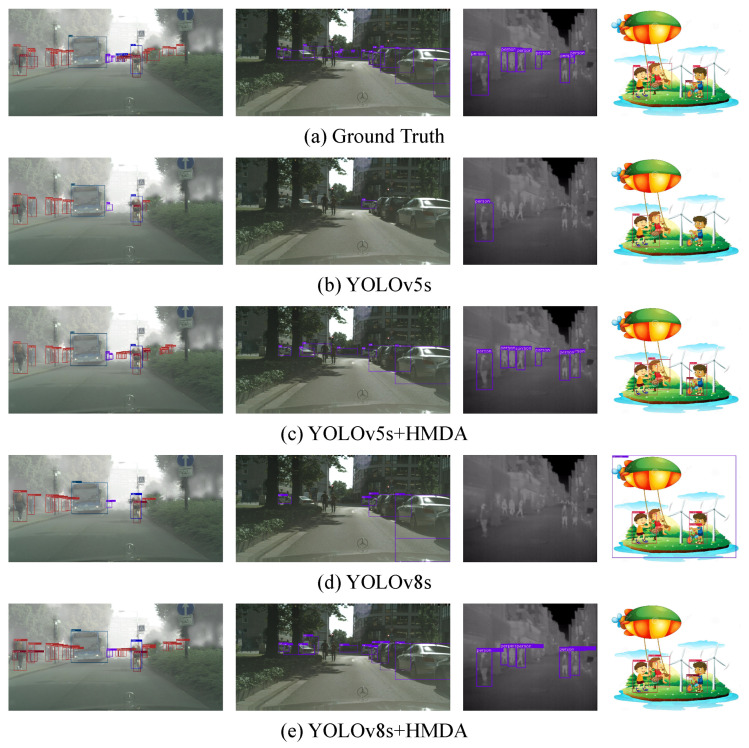
Illustration of the detection results on the target domain. From left to right are results of C→F, S→C, KR→KL, and P→Cl, respectively. (**a**–**e**) denote the groud truth, YOLOv5, YOLOv5+HMDA, YOLOv8, and YOLOv8+HMDA (Zoom in for better view).

Figure 3 presents some object detection results of the ground truth, YOLOv5, YOLOv5+HMDA, YOLOv8, and YOLOv8+HMDA on tasks Cityscapes → Foggy Cityscapes, KAIST RGB → KAIST LWIR, Sim10k → Cityscapes, and Pascal VOC → Clipart. The proposed HMDA shows the better cross-domain detection performance compared with YOLOv5s and YOLOv8s.

#### 4.4.3. Convergence

**Figure 4:**
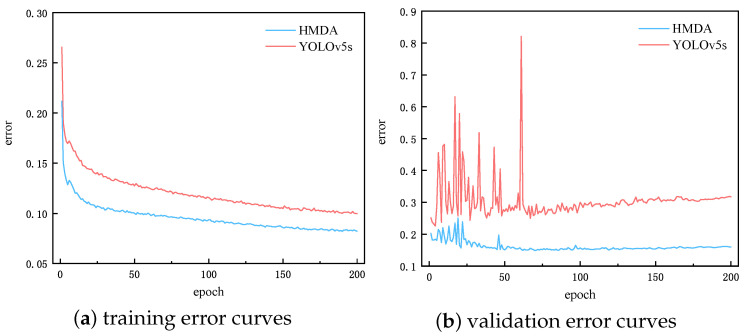
Training and validation error curves of YOLOv5s and HMDA-YOLO on task Cityscapes → Foggy Cityscapes.

The model’s convergence in the training stage is important for DAOD algorithms. We analyzed the convergence of YOLOv5s and HMDA-YOLO on task Cityscapes → Foggy Cityscapes, by recording the training and validation error curves (composed of $L_{cls}$. $L_{box}$, and $L_{obj}$) in Figure 4. It can be seen that the convergence trend of the source supervision is basically the same for both methods, but HMDA-YOLO can converge better. As for the target validation, the proposed method is obviously superior, with more stable convergence and less loss. In contrast, YOLOv5s has a progressively higher validation error without transfer learning strategy. The above analyses demonstrate that HMDA can converge faster and better, and can guarantee the performance of model on both source and target domain samples.

#### 4.4.4. Influence of IOU Threshold

**Figure 5:**
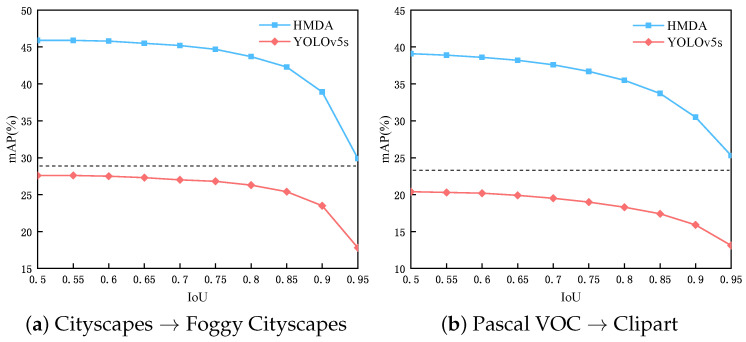
The performance with the variation in IOU thresholds on two tasks.

Figure 5 illustrates the performance of YOLOv5s and HMDA-YOLO on two different tasks as the IoU threshold changes from 0.5 to 0.95 (at intervals of 0.05). Generally, the overall detection accuracy is gradually decreasing as the IoU threshold increases. However, we have two insightful findings. The first one is that the YOLOv5-based method can localize the object accurately, which makes it possible to maintain good results when the threshold is increasing. The second point is that the proposed HMDA-YOLO greatly improves the cross-domain detection capability. And it still outperforms the baseline YOLOv5s with a IoU threshold of 0.5 even when the threshold of HMDA-YOLO is set to 0.95, which are separated by dash lines. The above findings demonstrate the effectiveness of the proposed HMDA-YOLO.

#### 4.4.5. Model Complexity

An important reason for choosing YOLO framework is its efficiency in real-time detection. Therefore, we analyzed the model complexity to show its advantage. The model’s parameters and inference time of different detectors are shown in Table 7. Compared to other architectures, YOLOv5s and YOLOv8s are light-weight, less complex, and more efficient. HMDA+YOLOv5s has about 12.9M parameters because of multiple domain discriminators during the training stage. In inference time, there is no need to load the weight of domain discriminators and vanilla YOLO with adaptive weight is used. The YOLO series framework has huge advantages in terms of both model parameter quantity and inference speed.

## 5. Conclusions

In this paper, we propose a novel DAOD method, HMDA-YOLO, to address the problem of domain shift in cross-domain object detection tasks. Considering various factors, such as performance, efficiency, and engineering practice, we adopted YOLOv5 as the baseline instead of the outdated Faster RCNN. The core of HMDA-YOLO is the hierarchical backbone adaptation and multi-scale head adaptation. The backbone adaptation is hierarchically performed at different levels depending on the level of feature abstraction, which promotes efficient adaptation and suppresses negative transfer. The head adaptation is adopted in three scales to enhance the model’s cross-domain performance and not impair its discriminative ability. The experimental results of multiple cross-domain tasks demonstrate that the proposed HMDA-YOLO is characterized by excellent cross-domain detection performance and fast detection speed.

## Figures and Tables

**Table 1:** The number of images in different domains.

Tasks	Training Set		Validation Set	Classes
	Source Domain	Target Domain	Target Domain	
C→F	Cityscapes	Foggy Cityscapes	Foggy Cityscapes	8
	2975	2975	500	
S→C	Sim10K	Cityscapes	Cityscapes	1
	10,000	2975	500	
KR→KL	KAIST RGB	KAIST LWIR	KAIST LWIR	1
	7601	7601	2252	
P→Cl	Pascal VOC	Clipart	Clipart	20
	16,551	500	500	

**Table 2:** Detection results (%) across different visibility (C→F).

Method	Car	Bicycle	Person	Rider	Mbike	Bus	Truck	Train	mAP
Faster RCNN	39.9	28.3	28.5	34.2	23.4	26.3	14.7	11.4	25.8
DAF	40.5	27.1	25.0	31.0	20.0	35.3	22.1	20.4	27.6
SWDA	43.5	35.3	29.9	42.3	30.0	36.2	24.5	32.6	34.3
ATF	50.0	38.8	34.6	47.0	33.4	43.3	23.7	38.7	38.7
HTCN	47.9	37.1	33.2	47.5	32.3	47.4	31.6	40.9	39.8
UMT	48.6	37.4	33.0	46.7	30.4	**56.5**	**34.1**	46.8	41.7
TIA	49.7	38.1	34.8	46.3	37.7	52.1	31.1	**48.6**	42.3
TDD	55.7	41.4	39.6	47.5	37.0	47.6	33.8	42.1	43.1
D-DETR	44.2	35.5	37.7	39.1	21.6	26.8	17.2	5.8	28.5
SFA	62.6	44.0	46.5	48.6	28.3	46.2	25.1	29.4	41.3
DA-DETR	63.1	**46.3**	43.5	50.0	31.6	45.8	24.0	37.5	26.3
YOLOv5s	46.9	29.7	35.8	37.9	15.4	26.7	11.5	6.6	26.3
S-DAYOLO	61.9	37.3	42.6	42.1	24.4	40.5	23.5	39.5	39.0
Confmix	62.6	33.5	45.0	43.4	28.6	45.8	27.3	40.0	40.8
**HMDA**	**66.1**	40.9	**49.1**	**51.6**	**38.4**	54.4	27.8	48.0	**45.9**
YOLOv8s	54.8	43.3	45.0	51.0	22.0	38.1	16.6	3.2	34.3
**HMDA**	72.1	49.8	54.6	60.4	40.9	60.9	35.9	60.8	54.4

**Table 3:** Detection results (%) from synthetic to real (S→C).

Method	mAP
Faster RCNN	34.6
DAF	38.9
SWDA	40.1
HTCN	42.5
UMT	43.1
D-DETR	47.4
SFA	52.6
DA-DETR	54.7
YOLOv5s	51.6
Confmix	56.3
**HMDA**	**58.6**
YOLOv8s	58.6
**HMDA**	64.8

**Table 4:** Detection results (%) across heterogeneous data (KR→KL).

Method	mAP
Faster RCNN	9.4
DAF	21.8
SWDA	31.3
YOLOv5s	5.5
**HMDA**	**45.4**
YOLOv8s	10.7
**HMDA**	33.8

**Table 5:** Detection results (%) across large domain shift (P→Cl).

Method	Aero	Bike	Bird	Boat	Bottle	Bus	Car	Cat	Chair	Cow	
Faster RCNN	35.6	52.5	24.3	23.0	20.0	43.9	32.8	10.7	30.6	11.7	
DAF	15.0	34.6	12.4	11.9	19.8	21.1	23.2	3.1	22.1	26.3	
SWDA	26.2	48.5	32.6	33.7	38.5	54.3	37.1	18.6	34.8	58.3	
HTCN	33.6	58.9	**34.0**	23.4	45.6	57.0	39.8	12.0	39.7	51.3	
ATF	41.9	**67.0**	27.4	**36.4**	41.0	48.5	42.0	13.1	39.2	**75.1**	
D-DETR	24.8	50.5	14.0	22.8	11.5	50.7	28.7	3.0	26.5	32.6	
SFA	35.2	47.6	33.5	38.3	39.6	40.4	38.5	**27.2**	37.6	43.1	
DA-DETR	**43.1**	47.7	31.5	33.7	21.4	62.8	42.6	14.8	39.5	44.2	
YOLOv5s	12.8	39.7	8.2	11.5	34.0	33.4	19.5	0.7	34.4	6.3	
**HMDA**	22.8	58.6	23.0	18.2	**50.3**	**70.5**	**44.7**	12.8	**45.1**	44.0	
YOLOv8s	22.4	60.1	27.5	33.2	44.0	37.9	23.5	10.1	57.2	7.8	
**HMDA**	26.3	66.8	28.1	36.6	55.3	65.1	36.5	13.6	60.1	51.8	
**Method**	**Table**	**Dog**	**Hrs**	**Mbike**	**Prsn**	**Plnt**	**Sheep**	**Sofa**	**Train**	**TV**	**mAP**
Faster RCNN	13.8	6.0	36.8	45.9	48.7	41.9	16.5	7.3	22.9	32.0	27.8
DAF	10.6	10.0	19.6	39.4	34.6	29.3	1.0	17.1	19.7	24.8	19.8
SWDA	12.5	12.5	33.8	65.5	54.5	52.0	9.3	24.9	54.1	49.1	38.1
HTCN	20.1	20.1	39.1	72.8	61.3	43.1	19.3	30.1	50.2	51.8	40.3
ATF	33.4	7.9	**41.2**	56.2	61.4	50.6	**42.0**	25.0	53.1	39.1	**42.1**
D-DETR	22.1	17.4	19.6	**73.1**	54.2	20.8	11.5	12.6	55.2	30.3	29.1
SFA	23.9	31.6	32.5	72.5	**66.8**	43.0	18.5	29.0	53.0	44.9	39.8
DA-DETR	**35.9**	**27.5**	31.8	72.6	65.6	42.2	17.3	31.1	**71.3**	50.1	41.3
YOLOv5s	8.0	10.1	17.0	8.9	28.6	45.7	2.5	20.4	24.3	42.5	20.4
**HMDA**	15.8	10.0	22.6	12.8	52.3	**55.6**	5.7	**36.7**	31.3	**61.3**	39.1
YOLOv8s	28.5	14.8	39.7	44.8	44.6	58.7	15.8	19.8	29.1	54.8	33.7
**HMDA**	32.6	12.5	38.1	50.6	59.6	64.7	28.7	34.1	34.8	60.5	42.8

**Table 6:** Ablation study (%) of HMDA-YOLO based on task C→F.

Method	Car	Bicycle	Person	Rider	Mbike	Bus	Truck	Train	mAP
YOLOv5s	46.9	29.7	35.8	37.9	15.4	26.7	11.5	6.6	26.3
Base + $L_{sa}$	52.0	38.7	42.2	47.4	29.3	37.6	21.2	15.6	35.5
Base + $L_{ma}$	60.3	39.5	45.0	48.5	26.0	39.3	23.9	35.3	39.7
Base + $L_{da}$	59.2	40.9	46.0	48.4	31.4	37.6	22.5	17.6	37.9
Base + $L_{backbone}$	64.4	40.5	48.2	51.2	33.3	44.9	25.0	41.8	43.7
Base + $L_{head}$	59.7	45.0	47.3	50.3	30.2	38.6	24.3	23.7	39.9
HMDA-YOLO	**66.1**	**40.9**	**49.1**	**51.6**	**38.4**	**54.4**	**27.8**	**48.0**	**45.9**

**Table 7:** Model complexity of different detectors.

Detector	Faster RCNN	Faster RCNN	D-DETR	YOLOv5s	YOLOv8s
Backbone	VGG16	ResNet50	ResNet50	CSPDarknet	CSPDarknet
Params (M)	138.4	25.6	40.2	7.2	11.2
Inference Time (FPS)	5	7–15	19	95	100

## Data Availability

The data used in the experiment can be obtained at: https://www.cityscapes-dataset.com/, http://host.robots.ox.ac.uk/pascal/VOC/, https://github.com/naoto0804/cross-domain-detection/tree/master/datasets, https://soonminhwang.github.io/rgbt-ped-detection/, and https://fcav.engin.umich.edu/projects/driving-in-the-matrix (accessed on 12 July 2025).
